# TongueNet-GYN: a multimodal deep learning framework for non-invasive gynecological disease screening in digital public health

**DOI:** 10.3389/fpubh.2026.1854215

**Published:** 2026-06-23

**Authors:** Chang Liu, Yuwei Luo, Tianzi Chen, Jie You, Jinping Wang

**Affiliations:** 1 Department of Hospitality and Business Management, Technological and Higher Education Institute of Hong Kong, Hong Kong, China; 2 School of Advanced Engineering, Great Bay University, Dongguan, China; 3 Basic Medical School of Naval Medical University, Naval Medical University, Shanghai, China; 4 Department of Information and Software Engineering, East China JiaoTong University, Nanchang, China; 5 Department of Traditional Chinese Medicine, Jiangsu Kunshan Hospital of Integrated Traditional Chinese and Western Medicine, Kunshan, China

**Keywords:** chronic disease management, gynecological diseases, health equity, multimodal fusion, non-invasive screening

## Abstract

**Background:**

Gynecological diseases, such as polycystic ovary syndrome (PCOS) and endometriosis, are prevalent global health concerns. Conventional diagnostics often rely on invasive procedures or costly imaging, limiting accessibility in resource-constrained settings. This study proposes TongueNet-GYN, a novel, non-invasive screening framework that leverages tongue image analysis integrated with modern AI.

**Methods:**

We compiled a dataset of 3,167 tongue images. To address class imbalance, a hybrid strategy combining Borderline-SMOTE and clinically constrained data augmentation was employed. The framework integrates structured clinical priors with deep semantic features extracted via an enhanced Attention-CLIP model. Additionally, quantified morphological features were incorporated to mirror clinical diagnostic logic.

**Results:**

TongueNet-GYN was evaluated using a robust framework comprising 5-fold cross-validation on a discovery set (85%) and subsequent validation on an independent held-out test set (15%). The model achieved a high diagnostic Accuracy of 90.14% and an AUC of 89.74% on the unseen test data. Furthermore, the integration of patient age was identified as a critical factor, yielding measurable improvements in both diagnostic accuracy and framework robustness

**Conclusion:**

These results demonstrate that TongueNet-GYN provides a precise, efficient, and scalable digital health solution, offering potential for improving early screening and health equity in women's chronic disease management.

## 1 Introduction

Gynecological diseases, such as polycystic ovary syndrome (PCOS) and endometriosis, represent a significant and prevalent health burden for women globally (1). Epidemiological data indicate that such conditions affect a substantial portion of the female population, with PCOS alone having an estimated prevalence of up to 10–13% among reproductive-aged women. The impact of these disorders extends beyond reproductive health, often leading to long-term metabolic complications, psychological distress, and a diminished quality of life. The economic burden is also considerable, encompassing direct healthcare costs and indirect costs from lost productivity (2). Consequently, the early and accurate detection of gynecological diseases is of paramount clinical importance. Timely intervention has been demonstrated in numerous studies to significantly improve patient outcomes, prevent disease progression, and reduce associated complications (3).

A common diagnostic approach for gynecological conditions, such as polycystic ovary syndrome (PCOS) and ovarian dysfunction, involves pelvic ultrasonography and serum hormone level testing, as recommended by leading endocrinology and gynecology societies. This technique employs a multifaceted evaluation that takes into account factors like follicular count and ovarian volume via ultrasound, alongside hormonal markers such as testosterone, LH, and FSH levels. The risk of long-term metabolic syndromes increases significantly (HR = 2.87, 95% CI: 2.10–3.92) (5) and the prevalence of infertility rises by 2.1 times when diagnostic criteria are fully met, according to numerous clinical cohorts (6). These conventional diagnostic methods, however, have several drawbacks in real-world clinical settings. First, the interpretation of ultrasound images, particularly the assessment of ovarian morphology and endometrial thickness, heavily relies on the subjective judgment and experience of sonographers. There is only a medium level of consistency between different operators (k = 0.52–0.65) (4). Second, these techniques have limitations in certain contexts. For instance, obtaining clear ultrasound images can be challenging for patients with obesity or anatomical anomalies, and hormone levels can be skewed by transient factors like acute stress or the use of certain medications. Additionally, in primary care or resource-limited settings, access to high-resolution ultrasound machines and reliable hormone assay facilities is often limited, which makes comprehensive screening challenging to conduct. According to data from a multi-center survey, only about 15% of primary care clinics in rural areas are equipped to perform such diagnostic workflows, and the average waiting time for a specialist diagnosis can be 4.2 ± 1.8 weeks. In regions with scarce medical resources, the accessibility is significantly lower. As a result, there is a high demand for diagnostic methods that are precise, efficient, and accessible for gynecological health screening (7).

Recent advances in image processing and machine learning have opened up new avenues for non-invasive diagnostic methods (20). In addition to reflecting a wide range of physiological and pathological states, the human tongue is a valuable non-invasive diagnostic tool. According to studies, tongue image analysis holds great promise for the identification of diseases, and there is a strong correlation between the morphological features of the tongue and general health. For instance, a study by Jiang et al. (23) analyzed the tongue features of women with menstrual disorders and found that specific manifestations, such as pale tongue color and thin white coating, were significantly associated with patterns of Blood deficiency in Traditional Chinese Medicine, which underlies various gynecological conditions (31). The Khalid Al-hammuri team (8) used the Vision Transformer to analyze the color spatial characteristics of HSV on the tongue surface during the COVID-19 epidemic (9). They discovered that the diagnostic accuracy rate was 85.7% and that the saturation (S value) of the anterior area of the tongue decreased by 15 ± 3% (*p* < 0.01) in virus-infected individuals. With a diabetes identification accuracy rate of 92.3% in 10,284 samples, the Deep Tongue system created by Zhao et al. (16) using the ResNet-101 architecture outperformed the conventional screening method based on fasting blood glucose (AUC = 0.81, *p* < 0.001). This technique has a sensitivity of 89.5% and is linked to microvascular lesions by detecting the texture features of a yellow and oily coating on the tongue surface (GLCM contrast > 35) (10). The objective of this research project is to create a machine learning-based diagnostic system that can use tongue pictures to predict Gynecological Disease. By synergizing multimodal features—including static clinical indicators and high-level semantic embeddings—while integrating patient age as a key demographic covariate, we aim to enhance the diagnostic accuracy and clinical utility of gynecological disease risk assessment (11).

To fully capture the multi-dimensional characteristics of tongue images, we developed a multimodal feature fusion mechanism. This architecture effectively integrates high-level semantic visual features with low-level detailed morphological information, enhancing the accuracy and robustness of tongue image analysis. Specifically, we leverage an EA-CLIP (enhanced Attention-CLIP) model as the core feature extractor. By combining the CLIP framework with an attention mechanism (12), our model demonstrating competitive performance relative to established benchmarks in the field. Furthermore, to address the challenge of class imbalance in medical datasets, this study introduces a hybrid learning framework integrating Borderline-SMOTE and clinically-constrained data augmentation (13). It is essential to emphasize that our augmentation strategy is strictly governed by clinical diagnostic priors: through HSV color-space decoupling, we strictly preserved the Hue channel to avoid distortion of pathological color features, while allowing modest adjustments to Value and Saturation to simulate realistic variations in illumination and tissue hydration. Simultaneously, rotational transformations within ±15 were introduced to ensure the model's robustness against tongue positioning variability. To uphold medical ethical integrity, Borderline-SMOTE was executed exclusively within the latent feature space rather than the image space; this approach effectively densifies the distribution of minority samples at the decision boundary while avoiding the generation of anatomically unreliable synthetic images (14). By focusing the loss function on minority samples and enriching their distribution. This multi-stage methodology directs the loss function to focus on hard-to-classify samples, enhancing both diagnostic sensitivity and precision (15).

The main contributions of this study are summarized as follows:

(1) Novel Multimodal Fusion Framework for Gynecological Screening, the multimodal approach bridges the gap between holistic tongue diagnosis and objective clinical data, achieving superior screening performance compared to single-modality baselines.
(2) Clinically Constrained Learning with Ethical Integrity: Implemented a balancing framework using latent-feature SMOTE and clinical-prior augmentations. This ensures high sensitivity while strictly preserving pathological authenticity and ethical integrity.
(3) EA-CLIP: We propose a novel architecture featuring dual visual encoders to concurrently capture global semantic contexts and localized morphological details of tongue images. By integrating a bi-directional attention mechanism, the model adaptively aligns multi-source features, establishing a rigorous mathematical correlation between pathological patterns and clinical risks while maintaining high diagnostic transparency.

## 2 Methods

### 2.1 Framework architecture

**Figure 1:**
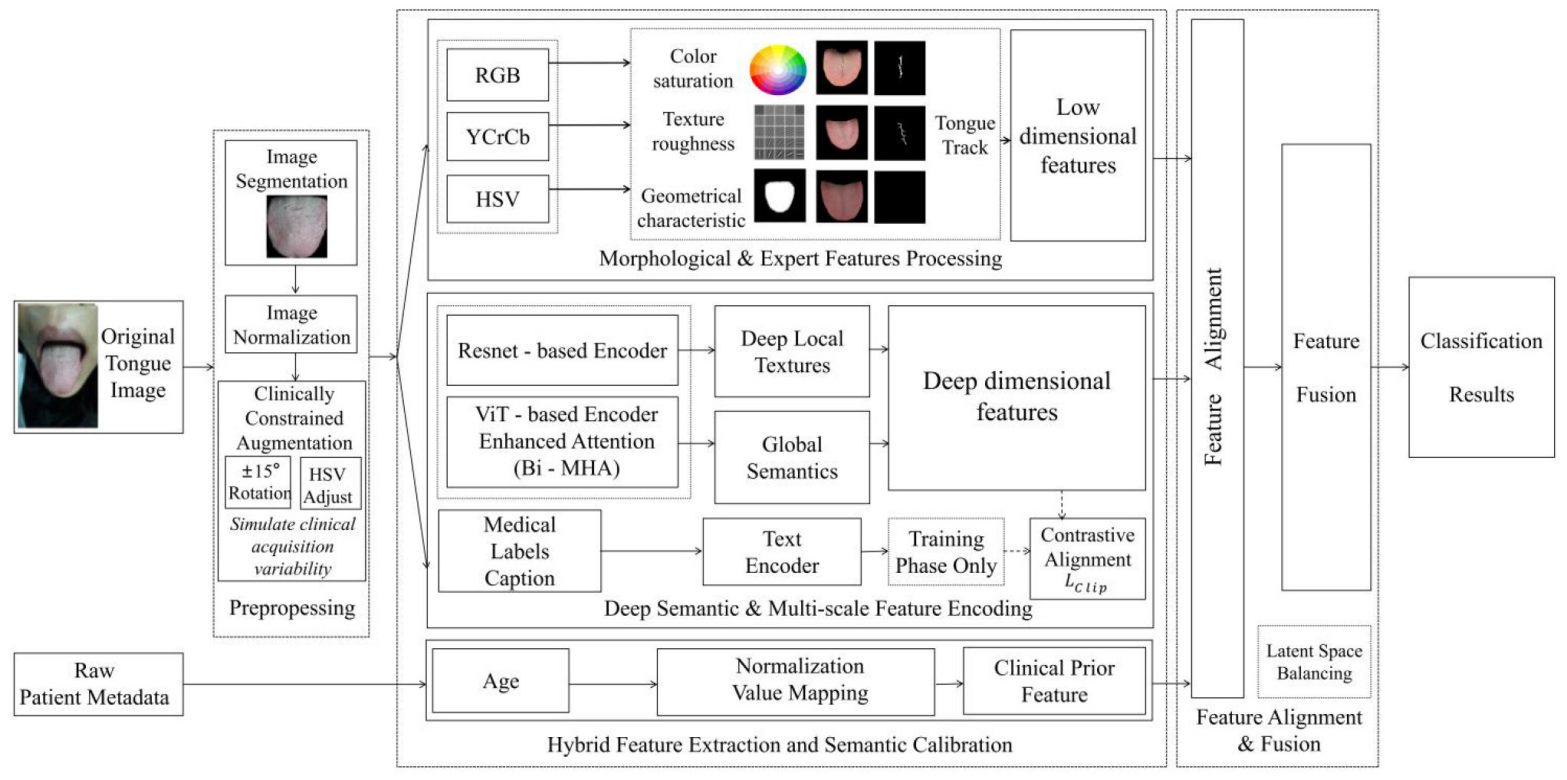
Overall framework.

Our gynecological disease diagnosis system based on analysis of tongue images is founded upon an information fusion paradigm with integration of manually extracted static features and encoded automatic features. The overall framework has parallel feature extraction branches of complementary features of the tongue images. One is assigned to the extraction of manually crafted static features—covering such properties as coloration of tongue, thickness of coating, color of coating, crack presence on the tongue, and fissure size—whereas the other uses a EA-CLIP framework trained previously to generate high-dimensional image embeddings (12). The two sets of features, coupled with the age of the patient, are combined linearly to create a holistic feature vector that is finally used to train a SVM classifier for predicting the risk of gynecological diseases. A diagram representing the holistic framework is presented in Figure 1.

### 2.2 Segmentation of the tongue picture

The inherent intricacy of facial backgrounds found in original clinical images requires the accurate definition and segmentation of the tongue area with the aim of reducing the potentially misleading impact of extraneous anatomical information on further diagnostic assessments (27). The accuracy of the segmentation process is, in fact, a determinant of computer-based disease diagnosis system validity. Advances in automatic segmentation processes in recent times have spurred universal implementation of deep learning architectures, particularly the U-Net architecture, which has achieved outstanding outcomes in the analysis of biomedical images. It should be noted that the nnU-Net variant incorporates adaptive learning rate modifications as well as class balancing techniques that offer improved segmentation outcomes as evidenced by comparative evaluations (17).

Methodologically, we used Labelme software (version 5.2.1) to conduct expert-guided manual annotation on a clinical tongue image dataset of 3,167 images to get accurate delineation of tongue contours. We used the Adam optimization algorithm to optimize the network, setting the hyperparameters as β1 = 0.9 and β_2_ = 0.99, and trained the model at an input resolution of 512 × 512 pixels with a batch size of 32 for 50 epochs. For stabilizing training, we used cosine annealing learning rate scheduling with the initial learning rate as 1 × 10^4^, and used transfer learning by initializing model parameters trained from the Carvana dataset. On testing the model, we performed comparative analysis of four segmentation models: Snake model, FCN, U-Net, and nnUNet. The segmentation performance was rigorously evaluated using 5-fold cross-validation to ensure the stability and generalizability of the proposed framework. For each fold, the optimal hyperparameter configuration was applied, and the final results are reported as the mean ± standard deviation across four key metrics: Dice Coefficient (DC), Recall, Precision, and F1-score. As illustrated in Table 1, the nnUNet architecture demonstrated near-perfect performance. The minimal standard deviations across all metrics statistically validate the high consistency and effectiveness of the framework in accurately delineating tongue tissue within complex facial images, confirming that the performance is statistically significant and robust against data variability.

**Table 1:** Performance comparison of three segmentation algorithms.

Methods	Evaluation stage	Dice coefficient (%)	Recall (%)	Precision (%)	F1-score (%)
Snake	5-Fold CV (Mean ± SD)	58.82 ± 3.45	63.30 ± 4.12	65.67 ± 3.88	64.48 ± 4.01
	Test (95% CI)	59.12 [58.4, 59.8]	63.55 [62.7, 64.4]	65.90 [65.1, 66.7]	64.72 [63.9, 65.5]
U-net	5-Fold CV (Mean ± SD)	91.85 ± 1.22	91.45 ± 1.45	94.60 ± 0.98	93.02 ± 1.15
	Test (95% CI)	91.57 [91.1, 92.6]	91.20 [90.6, 92.3]	94.32 [93.9, 95.3]	92.57 [91.3, 92.9]
FCN	5-Fold CV (Mean ± SD)	93.88 ± 1.05	96.80 ± 0.88	95.25 ± 1.12	92.10± 1.34
	Test (95% CI)	93.52 [93.2, 94.6]	96.52 [96.2, 97.4]	95.00 [94.5, 96.0]	91.57 [91.3, 92.9]
**nnUNet**	5 - Fold CV (Mean ± SD)	**99.72** **±0.06**	**99.72** **±0.05**	**99.75** **±0.04**	**99.71** **±0.06**
	Test (95% CI)	**99.79 [99.76, 99.82]** ^*^	**99.83 [99.80, 99.86]** ^*^	**99.89 [99.87, 99.91]** ^*^	**99.76 [99.74, 99.88]** ^*^

Performance metrics are presented across two tiers: 5-Fold CV (Mean ± SD) represents the average stability within the discovery set, while Test (95% CI) represents the final evaluation on the independent held-out set. The 95% Confidence Intervals (CI) were derived via Bootstrap resampling (N = 1000) to quantify the statistical reliability of the metrics. Statistical significance is indicated by ^*^p < 0.05 (paired *t*-test) compared to the different models. Bold values indicate the best performance.

### 2.3 Feature extraction

Feature extraction is used both as a term for low-dimensional feature extraction and high-dimensional feature extraction. Manual Feature Extraction: After being manually segmented with caution, each tongue image is converted to alternative color spaces in order to obtain essential diagnostic information. In our methodological framework, we extract the following static features: Tongue Color: Measured in terms of the CIELAB color space to identify subtle hue differences that may reflect deficiencies. Coating Thickness and Coating Color: Using image processing techniques, we measure the relative coating thickness and whether it is yellowish or white. Tongue Cracks and Fissure Area: Using edge detection and morphological processing, we enable quantification of the cracks and measurement of the fissure area, which are indirect measures of systemic health. Additional Feature is the patient age. Age is incorporated into the feature vector, acknowledging its influence on hormonal levels or gynecological health status (19).

Deep Feature Extraction: In order to adequately capture the dense, high-level semantic information embedded in tongue images, we use a pretrained Contrastive Language–Image Pre-training (CLIP) model. The CLIP model, pre-trained on a large image-text pair dataset, generates a strong embedding that encompasses rich visual patterns as well as subtle color and texture variations that manual methods normally fail to capture. These embeddings, conventionally represented as high-dimensional vectors, are utilized to supplement the manually extracted features and boost the model's overall discriminative capability.

**Figure 2:**
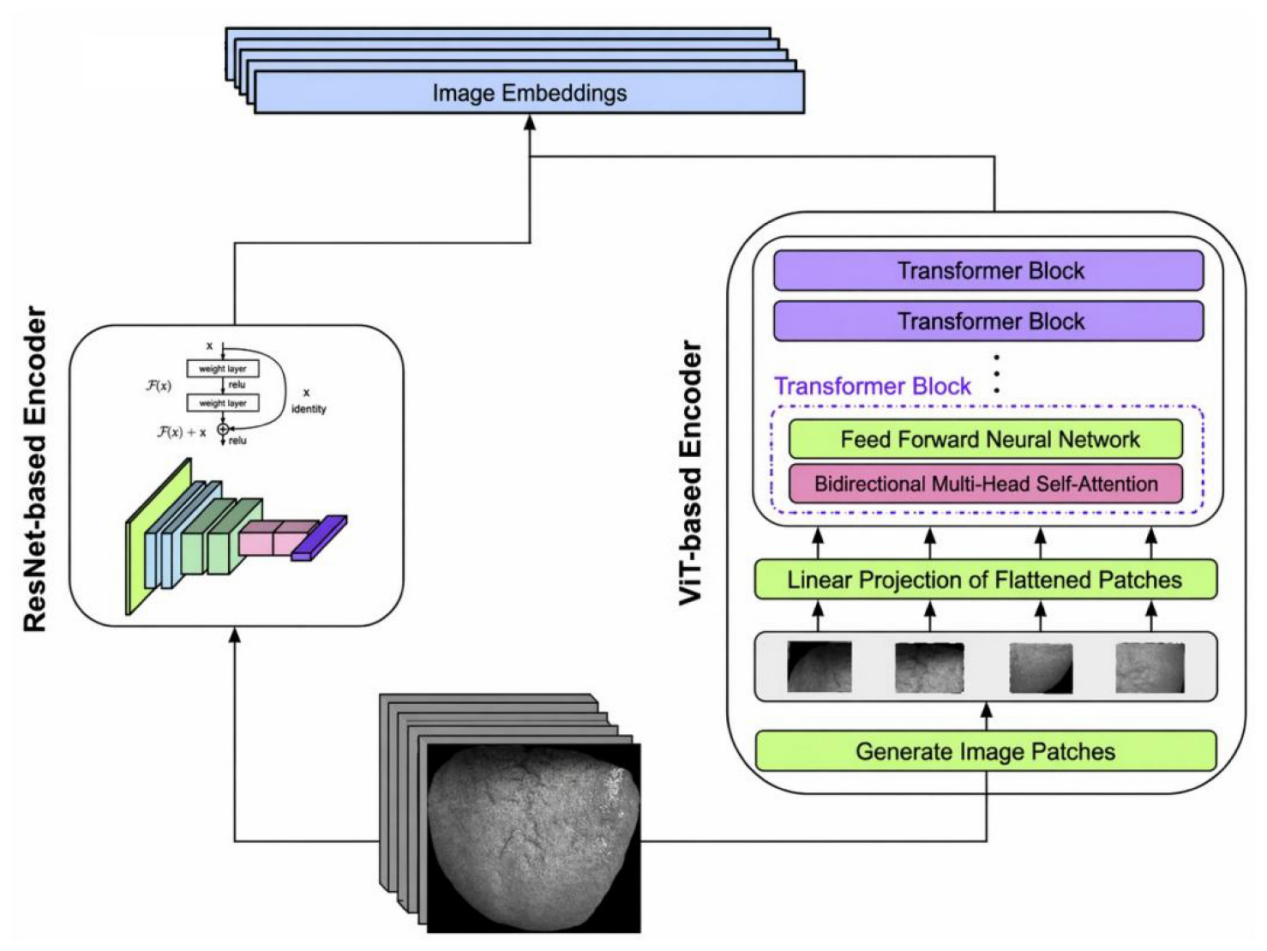
Complete detailed demonstration of CLIP high-dimensional feature extraction.

Distinct from the standard CLIP framework, our proposed architecture employs a Dual-Visual-Encoder strategy to achieve a more granular characterization of tongue features (18). As shown in Figure 2, the suggested architecture for visual input processing utilizes a transformer-based image embedding architecture and a Resnet-based encoder (25–28). This design integrates a Transformer-based encoder for capturing global semantic relationships and a ResNet-based CNN encoder for extracting local morphological details (29, 30). By mapping these complementary embedding vectors into a unified latent space via contrastive learning, the model achieves superior alignment with clinical text embeddings. This dual-pathway approach significantly enhances the robustness of feature extraction, ensuring that both the holistic tongue structure and subtle pathological textures are adequately representedFor the transformer-based image embedding architecture, first, a patch generation module divides the input images into non-overlapping patches. Then, the patches are linearly embedded into a flattened embedding space to create the input sequence for sophisticated processing. To find long-range correlations between the image patches, bidirectional multi-head self-attention mechanisms are incorporated into each of the several stacked transformer blocks constituting the base architecture. Next, a feed-forward neural network processes the feature representations. Since the model has a hierarchical structure, it is able to accumulate progressively both global and local visual information and retain spatial relationships between the image (24).

The architecture implies processing visual information by a sequence of operations. Given an input image is shown in Formula (1)


$$\begin{array}{c} \text{I}\in {\mathbb{R}}^{\text{H}\times \text{W}\times \text{C}} \end{array}$$ (1)

In Formula (2), then the input image is partitioned into N non-overlapping patches:


$$\begin{array}{c} \text{P}\in {\mathbb{R}}^{\text{N}\times \text{(}{\text{p}}^{2}\text{C)}} \end{array}$$ (2)

In which is (3):


$$\begin{array}{c} \text{N =}\frac{H}{P}\times \frac{W}{P} \end{array}$$ (3)

Then each patch is projected to D dimensions via linear transformation in (4):


$$\begin{array}{c} {\text{Z}}_{\text{0}}\text{=}\left[{\text{X}}_{\text{p}}^{\text{1}}\text{E; }{\text{X}}_{\text{p}}^{\text{2}}\text{E;}...\;...\text{; }{\text{X}}_{\text{p}}^{\text{N}}\text{E}\right]\text{+}{\text{E}}_{\text{pos }} \end{array}$$ (4)

In which:


$$\begin{array}{c} \text{E}\in {\mathbb{R}}^{\left\{\left(p^{2}\;C\right)\times D\right\}} \end{array}$$ (5)

$$\begin{array}{c} E_{pos}\in {\mathbb{R}}^{\text{N}\times \text{D}} \end{array}$$ (6)

As shown in Formulas (5) and (6), by using the patch generation module, the aforementioned procedures decompose the input image into non-overlapping patches and project the tiny blocks into a flat embedding space in a linear fashion. The transformer module then receives the linearly mapped vectors. Each transformer block implement, In Formulas (7) and (8), *LN* stands for Layer Normalization: This is a standard normalization strategy in training that is used to stabilize and speed deep neural networks. This is accomplished by normalizing the activation of each sample rather than each batch, as in batch normalization.


$$\begin{array}{c} z_{\text{l}}^{\prime }\;=\text{ MHA (LN(}{\text{z}}_{l-1}))+z_{l-1} \end{array}$$ (7)

$$\begin{array}{c} z_{l}\;=\;FFN\;\left(LN\left({z_{l}}^{\prime }\right)\right)+\;{z_{l}}^{\prime } \end{array}$$ (8)

where MHA denotes Multi-Head Attention, as shown in Figure 2. It can be represented as a multi-head representation generated by different linear changes of the input query, key, and value. This step is usually achieved through different weight matrices. Among them, X is the input feature matrix, *W*^*K*^, *W*^*Q*^, *W*^*V*^ are the learnable weight matrices. As shown in Formula (9)


$$\begin{array}{c} Q=XW^{Q},\;K=XW^{K},\;V=XW^{V} \end{array}$$ (9)

To avoid excessively large dot product values, each attention head calculates the dot product of the query and the key, then divides it by $\sqrt{d_{k}}$ for scaling, which can keep the model's results steady during the training process.


$$\begin{array}{c} \text{Attention (Q, K, V) = softmax}\left(\frac{\text{Q}{\text{K}}^{\text{T}}}{\sqrt{{\text{d}}_{\text{k}}}}\right)V \end{array}$$ (10)

The output $z_{l}^{\prime }$ produced by this step is ultimately represented by Formula (10), which is an improved representation that combines the initial data with the extra data acquired by the attention mechanism. In addition to preserving the original input's information, adding the output to the original input *z*_*l*−1_ through residual connection integrates the sequence's complex relationships and context information, effectively resolving the issue of vanishing gradients in deep networks and supplying richer and more valuable features for the model's later layers.

The Feed-Forward Network (FFN) is the fundamental structure in deep learning (21, 22). Through a sequence of fully connected layers, non-linear transformations apply to the input data in order to acquire intricate feature representations and improve the model's expressiveness and prediction accuracy. Following multi-head attention mechanism processing, the output ${z_{l}}^{\prime }$ will go to the feedforward network (FFN) for additional non-linear modification. Formula (11) illustrates how this process works. The output ${z_{l}}^{\prime }$ is fed into the feedforward neural network with non-linear activation functions after it has undergone another round of re-normalization (LN) processing. Independent non-linear variations improve the model's capacity to manage intricate features. The feedforward network's computational steps can be summed up as follows, *W*_1_ and *W*_2_ are, respectively learnable weight matrices, while *b*_1_ and *b*_2_ are bias terms.


$$\begin{array}{c} FFN\left(x\right)=GELU\left(xW_{1}+b_{1}\right)W_{2}+b_{2} \end{array}$$ (11)

The final representation after *LN* blocks is shown in formula (12), *z*_*L*_ represents a feature vector mapping based on the VIT encoder.


$$\begin{array}{c} z_{L}=FFN\left(LN\left(z_{L-1}\right)\right) \end{array}$$ (12)

Following the generation of feature vector mappings using Vision Transformer (ViT), our model also employs ResNet as an alternative image embedding framework to produce corresponding feature mappings. ResNet adeptly captures the hierarchical features of images through its sophisticated deep convolutional neural network architecture. Notably, ResNet leverages residual connections to address the challenge of vanishing gradients that often occur during the training of deep networks. This innovative approach empowers the network to learn more nuanced and abstract feature representations.

The feature vectors derived from Resnet-based encoder are not only rich in the local details of the image but also encapsulate profound semantic insights. By incorporating both ViT-based and Resnet-based architectures, our model gains the capability to comprehend and represent image content from diverse viewpoints, yielding a wealth of image embedding vectors. These vectors are subsequently projected into a unified vector space to facilitate cross-modal alignment and matching. This dual-architecture strategy equips the CLIP model with the flexibility to process and interpret image data effectively, establishing a robust foundation for a variety of subsequent tasks, including image-text alignment and retrieval. Through contrastive learning, the model cultivates an embedding space where semantically correlated images and texts are drawn closer together, thus fostering efficient cross-modal interaction and comprehension.

In Formula (13), following the encoder's feature extraction, the framework incorporates Contrastive Language-Image Pretraining (CLIP) to enhance cross-modal representation learning. Given the extracted visual features $z_{L}\in {\mathbb{R}}^{N\times D}$ from the ViT and ResNet backbone, CLIP projects them into a joint embedding space through:


$$\begin{array}{c} {\text{v}}_{i}=W_{V}\;\cdot \text{Pool}\left({\text{z}}_{\text{L}}\right)+{\text{b}}_{\text{v}} \end{array}$$ (13)

where $W_{v}\;\in \;{\mathbb{R}}^{D\times d}\;$is a learnable projection matrix, Pool(z_L_) denotes global average pooling, and ${\text{v}}_{\text{i}}\;\in \;{\mathbb{R}}^{d}$ represents the final image embedding. The pooling operation aggregates the spatial information into a compact representation is shown in (14), where ${\text{z}}_{\text{L}}^{\left(i\right)}$ denotes the *i*−*th* feature token.


$$\begin{array}{c} \text{Pool}\left({\text{z}}_{L}\right)=\frac{1}{\text{N}}\overset{\text{N}}{\underset{i=1}{\sum }}{\text{z}}_{\text{L}}^{\left(\text{i}\right)} \end{array}$$ (14)

As shown in Formula (15), to align visual and textual modalities, CLIP employs a contrastive learning objective that maximizes the similarity between matched image-text pairs while minimizing it for mismatched pairs. Given a batch of *M* image-text pairs ${\left\{\left(v_{t}^{\left(i\right)},\;t_{t}^{\left(i\right)}\right)\right\}}_{i=1}^{M}$, the similarity scores are computed using a cosine similarity metric:


$$\begin{array}{c} \text{s}\left({\text{v}}_{\text{t}},{\text{t}}_{\text{t}}\right)=\frac{{\text{v}}_{\text{t}}{\text{t}}_{\text{t}}}{||{\text{v}}_{\text{t}}||\;||{\text{t}}_{\text{t}}||} \end{array}$$ (15)

Where ${\text{t}}_{\text{t}}\;\in \;{\mathbb{R}}^{d}$ is the textual embedding obtained from the CLIP text encoder. In formula (16), the contrastive loss L_CLIP_ is then defined as a symmetric cross-entropy loss over the similarity scores:


$$\begin{array}{c} {\text{L}}_{\text{CLIP}}=\\ -\frac{1}{2\text{M}}\overset{\text{M}}{\underset{\text{i}=1}{\sum }}\left[\log\frac{\exp\left(\frac{\text{s}\left({\text{v}}_{\text{t}}^{\left(\text{i}\right)},{\text{t}}_{\text{t}}^{\left(\text{i}\right)}\right)}{\text{τ}}\right)}{\sum_{\text{j}=1}^{\text{M}}\exp\left(\frac{\text{s}\left({\text{v}}_{\text{t}}^{\left(\text{i}\right)},{\text{t}}_{\text{t}}^{\left(\text{j}\right)}\right)}{\text{τ}}\right)}+\log\frac{\exp\left(\frac{\text{s}\left({\text{t}}_{\text{t}}^{\left(\text{i}\right)},{\text{v}}_{\text{t}}^{\left(\text{i}\right)}\right)}{\text{τ}}\right)}{\sum_{\text{j}=1}^{\text{M}}\exp\left(\frac{\text{s}\left({\text{t}}_{\text{t}}^{\left(\text{i}\right)},{\text{v}}_{\text{t}}^{\left(\text{j}\right)}\right)}{\text{τ}}\right)}\right] \end{array}$$ (16)

where τ is a temperature parameter that scales the logits.

The integration of a customized CLIP framework endows this study with superior cross-modal semantic alignment. Unlike the standard single-encoder paradigm, our Dual-Visual-Encoder architecture enables a more granular mapping between clinical linguistic descriptions and multifaceted tongue features. By optimizing a contrastive loss function, the model achieves effective decoupling within the feature space, allowing it to integrate medical prior knowledge while capturing both holistic chromatic patterns and subtle textural variations. This specialized design not only enhances the model's interpretative depth, also maintains high computational efficiency, as the dual-pathway feature extraction remains a streamlined forward process. The text embeddings used for CLIP contrastive learning were derived from standardized clinical descriptors but were not directly fed into the classifier; they served only to align the visual feature space during training.

### 2.4 Feature Fusion and Classification

The low - dimensional features, deep feature embeddings, and clinical feature are concatenated to construct a unified feature vector. This linear fusion ensures that both low- level and high-level information contribute to the prediction task. The fusion process is formalized in formula (17)


$$\begin{array}{c} \text{F=α}\cdot \;{\text{F}}_{static}\;⊕\beta \;\cdot {\text{F}}_{\text{CLIP}}\;⊕\;\gamma \;.A \end{array}$$ (17)

where F_static_ represents the vector of manually extracted features, F_CLIP_ denotes the deep features derived from the CLIP model, A is the patient age, and α, β, and γ are weighting coefficients set equally to ensure balanced contributions across features. This approach ensures that low-level morphological details and high-level semantic insights contribute equally to the initial joint representation, allowing the subsequent classifier to autonomously learn the optimal interactions between features without predefined heuristic bias.

For final diagnostic prediction, we strategically employ a Support Vector Machine (SVM) with a Radial Basis Function (RBF) kernel instead of a standard fully connected layer (MLP). This choice is dictated by SVM's superior generalization in clinical scenarios with high-dimensional features and a relatively limited sample size. Guided by the principle of Structural Risk Minimization, the SVM maximizes the classification margin, which inherently provides a more robust decision boundary and reduces the risk of overfitting compared to back-propagation neural networks. To ensure peak predictive stability, the SVM hyperparameters were rigorously optimized through a 5-fold cross-validation scheme on the discovery set. This iterative validation process ensures that the selected configuration captures the underlying patterns of gynecological risks across different data partitions. Detailed performance evaluation and statistical reliability, including 95% Confidence Intervals derived from Bootstrap resampling, are further presented in the Results section. The detailed comparative experiments in this section are presented and demonstrated in section 4.3 and section 5.2.

### 2.5 Dataset imbalance handling and loss function

The annotated dataset exhibits a moderate class distribution skew, with 1,905 negative and 1,262 positive samples. To mitigate potential bias toward the majority class and ensure robust feature representation for both categories, we implemented a synergistic strategy that integrates clinically-constrained data augmentation with contrastive representation learning. Rather than relying on simple oversampling, our approach focuses on enhancing the model's discriminative power through a multi-stage training process, preserving the diagnostic integrity of the original tongue images while optimizing performance across imbalanced classes.

Algorithm 1 Imbalanced data handling with multi-loss optimization. 
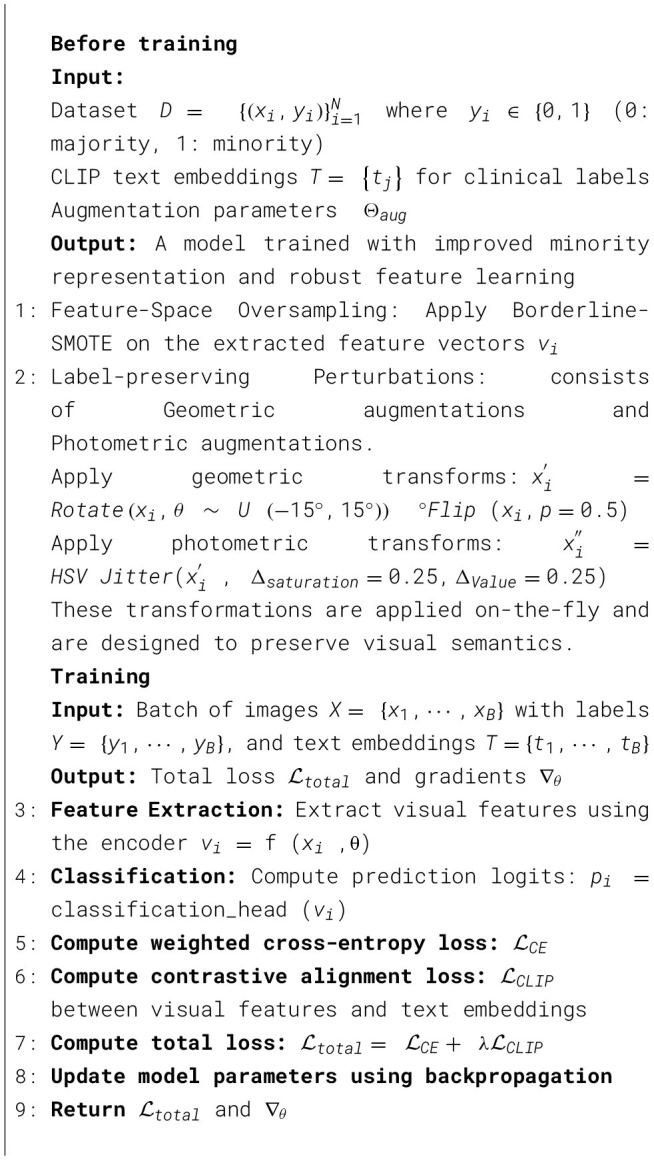

To avoid potential data leakage and ensure a fair evaluation, the dataset was first divided into training and testing subsets using sampling, preserving the original class distribution. Specifically, 85% of the data were used for training and 15% for testing. We adopt a feature-space re-weighting strategy based on Borderline-SMOTE. Specifically, minority samples located near class boundaries are interpolated in the learned feature space (k = 7 nearest neighbors) to adjust the effective class distribution. This operation is conducted on feature representations rather than raw images, and is used as a regularization mechanism to improve decision boundary learning, without introducing additional image-level samples. In parallel, we apply label-preserving perturbations during training to enhance robustness. These include mild geometric transformations and bounded photometric adjustments. All perturbations are applied on-the-fly and are designed to preserve visual semantics. The test set remains strictly untouched throughout the entire process and is used only for final evaluation.

To effectively address the class imbalance and enhance cross-modal feature alignment, we designed a multi-component loss function that integrates supervised classification with contrastive metric learning. The total loss $L_{total}$ consists of two key terms:

Cross-Entropy Loss ($L_{CE}$), which is the foundational classification loss, weighted to counteract class imbalance. Formula (18) provides clarification: ($W_{y_{i}}$ is the class weight, and *N* is the batch size).


$$\begin{array}{c} L_{CE}=-\frac{1}{N}\overset{N}{\underset{i=1}{\sum }}W_{yi}\left[y_{i}\log\left(P_{i}\right)+\left(1-y_{i}\right)\log\left(1-P_{i}\right)\right] \end{array}$$ (18)

CLIP Contrastive Loss ($L_{CLIP}$), which is designed to align visual features and text embeddings in a share space.

Finally, the expression of the loss function we obtain is the weighted sum of the two, as shown in Formula (19):


$$\begin{array}{c} {\text{L}}_{\text{total}}\;=\;{\text{L}}_{\text{CE}}+\lambda {\text{L}}_{\text{CLIP}} \end{array}$$ (19)

Where λ is a weighting coefficient that balances classification and alignment objectives. In our experiments, λ is empirically set to 0.2 based on validation performance. It inhibits the model's overfitting process while dynamically adapting.

## 3 Experiments

### 3.1 Dataset

In the current study, to ensure sufficient statistical power, we conducted a sample size calculation assuming a gynecological disease prevalence of 0.3 based on epidemiological data, with a target sensitivity of 0.8. At a 95% confidence level and a 5% confidence interval width, the minimum required sample size was determined to be 2,704 subjects. Ultimately, a total of 3,167 participants were enrolled in this prospective study at the Jiangsu Kunshan Hospital of Integrated Traditional Chinese and Western Medicine between March 2025 and July 2025. All participants were included according to predefined inclusion and exclusion criteria. The study protocol was strictly conducted in accordance with the Declaration of Helsinki and received formal approval from the Institutional Review Board (IRB) of Jiangsu Kunshan Hospital of Integrated Traditional Chinese and Western Medicine. To protect participant privacy, all clinical tongue images and associated diagnostic records were fully de-identified and anonymized at the source. Written informed consent was obtained from all participants. The final dataset comprises 3,167 standardized tongue images (1,905 healthy controls and 1,262 confirmed patients). To align visual features with clinical semantics, each positive sample was annotated with fine-grained labels derived from electronic medical records. Crucially, the Ground Truth for each image was not merely based on visual experience but established through a multi-modal synthesis of objective biomedical evidence. A panel of three senior experts adjudicated the labels by cross-referencing participants' biochemical profiles and pelvic ultrasound (USG) findings. Any diagnostic divergences were resolved by a third senior specialist via a formal adjudication mechanism. The detailed baseline characteristics, inclusion and exclusion criteria, and clinical prior distribution of the study cohort have been presented in Supplementary Table S1.

All images were captured under standardized lighting conditions to minimize variability. Preprocessing involved manual segmentation of the tongue region followed by normalization to standardize image dimensions and color intensity. Clinical diagnosis, confirmed through standardized gynecological examinations, served as the ground truth for each tongue image.

To ensure high-quality feature extraction, strict exclusion criteria were enforced to eliminate confounding factors such as pregnancy, acute infections, oral lesions, and dietary-induced tongue discoloration. The specific inclusion and exclusion criteria are detailed in Supplementary material. For binary classification, the dataset was divided using sampling into a training set (85%) and a dedicated test set (15%). To ensure rigorous optimization and prevent overfitting, a 5-fold cross-validation scheme was implemented within the training partition, where hyperparameters were fine-tuned to capture consistent pathological patterns across different data subsets.

### 3.2 Dataset augmentation

Dataset augmentation is a popular method in neural network learning that involves transforming the original dataset in a number of ways to increase its amount and diversity. This approach can lower the likelihood of overfitting while simultaneously enhancing the model's capacity for generalization. In this study, we particularly built a set of constrained data augmentation methods for the characteristics of tongue images in clinical practice, in addition to adopting generic data augmentation techniques, to improve the model's recognition capacity of tongue images.

**Figure 3:**
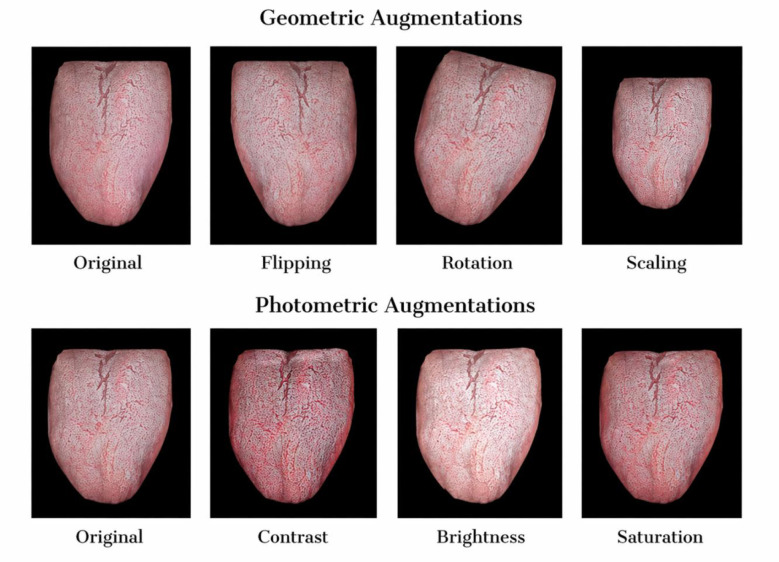
Illustrates examples of augmentation.

This method is tailored for the original positive samples and includes rotational transformations. This fluctuation range corresponds to variations in tongue position in clinical practice, as well as adjustments to the HSV color space (Value change Δ_Value_= 0.25, saturation change Δ_saturation_ = 0.25) to replicate various lighting situations. Strictly preserving the Hue channel to ensure that critical pathological color features remain unaltered. The abnormal color features are maintained. The goal of this augmentation method is to allow the model to adapt to numerous scenarios that may arise in the clinical environment, such as variable lighting conditions and variations in tongue position, hence boost the model's robustness and accuracy. This way, we can ensure that the model performs consistently in practical applications, even when confronted with photos that deviate somewhat from the training dataset. Figure 3 illustrates several examples of data augmentation techniques in this dataset. The entire processing procedure strictly adheres to clinical requirements and does not affect the original information density.

### 3.3 Implementation details

In this study, we employed a PyTorch-based framework to construct model architecture. PyTorch, a widely adopted tensor library in the domain of deep learning, facilitated our implementation. Training was executed on an NVIDIA Tesla V100 GPU, equipped with 32GB of memory, operating under a Windows 10 environment with CUDA version 10.1. Python, version 3.7.13, was the programming language of choice, and we leveraged the Scikit-Learn library to develop the machine learning model, while PyTorch was utilized for deep feature extraction. Throughout the training process, we adopted the Adam optimizer, an adaptive learning rate method that has shown remarkable performance in training deep learning models. The initial learning rate was set at 0.002, complemented by a weight decay of 0.0001 to mitigate overfitting. Additionally, we incorporated a cyclic learning rate strategy with a step size of 30 and a gamma value of 0.5, which aids the model in updating weights more efficiently during training. The training regimen comprised a total of 100 epochs, with a data augmentation probability of 0.1 to augment the diversity of the training dataset. The batch size for each domain was consistently set at 16, balancing memory consumption with the frequency of model updates. To further bolster the model's robustness against clinical noise, data augmentation was applied during each iteration with a probability of 0.1. This experimental setup ensures that the multi-modal features are effectively aligned and balanced before being fed into the optimized decision boundary.

### 3.4 Evaluation indices

In this study, the performance of classification was evaluated using the confusion matrix and other relevant indicators such as Accuracy (ACC) , Area under curve (AUC), Recall, and F1 index. In the binary classification task of tongue diagnosis, the predicted situations are usually divided into: True Positive (TP) and True Negative (TN), False Positive (FP), and False Negative (FN). Among them, True Positive (TP) represents the number of cases correctly predicted as positive, False Positive (FP) represents the number of cases wrongly predicted as positive, and True Negative (TN) represents the number of cases correctly predicted as negative, and False Negative (FN) represents the number of cases wrongly predicted as negative. The above four situations are acceptable. It is represented by the confusion matrix to evaluate the classification performance of the model. According to the confusion matrix, the following evaluation indicators can be calculated:

In Formula (20), Accuracy (ACC) is the fraction of correct prediction outcomes in the entire sample. The higher this indicator, the more accurate the model prediction and the lower the number of incorrectly predicted samples.


$$\begin{array}{c} ACC=\frac{TP+TN}{TP+FN+FP+TN} \end{array}$$ (20)

Specificity is often referred to as the True Negative Rate (TNR). As shown in Formula (21), out of the actually negative samples, this indicator shows the percentage of samples that were accurately anticipated to be negative. The misdiagnosis rate decreases with increasing specificity.


$$\begin{array}{c} Specificity=\frac{TN}{TN+FP} \end{array}$$ (21)

In Formula (22), sensitivity is often referred to as True Positive Rate (TPR) or recall. The percentage of samples that were accurately predicted to be positive among the actual positive samples is represented by this indicator. The missed detection rate decreases with increasing sensitivity.


$$\begin{array}{c} Sensitivity=\frac{TP}{TP+FN} \end{array}$$ (22)

As shown in Formula (23), The F1 score accurately reflects precision and sensitivity outcomes. The better the model's output result, the closer this index is to 1.


$$\begin{array}{c} \text{F}1=\frac{2\times \text{TPR}\times \text{PPV}}{\text{TPR}+\text{PPV}} \end{array}$$ (23)

Furthermore, the ROC curve is a curve plotted in terms of sensitivity and specificity, with TPR as the vertical coordinate and 1-TNR as the horizontal coordinate. The area under the ROC curve, or AUC value, can be used to assess the model's classification abilities. The model's performance and classification abilities improve as the AUC value increases. To ensure the statistical reliability of these high-performance results, 95% Confidence Intervals (CI) for all metrics were calculated using a Bootstrap resampling method (*n* = 1,000) on the independent test set, and consistent results across different data partitions were verified via 5-fold cross-validation.

## 4 Results

### 4.1 Classification results of the information fusion model

**Figure 4:**
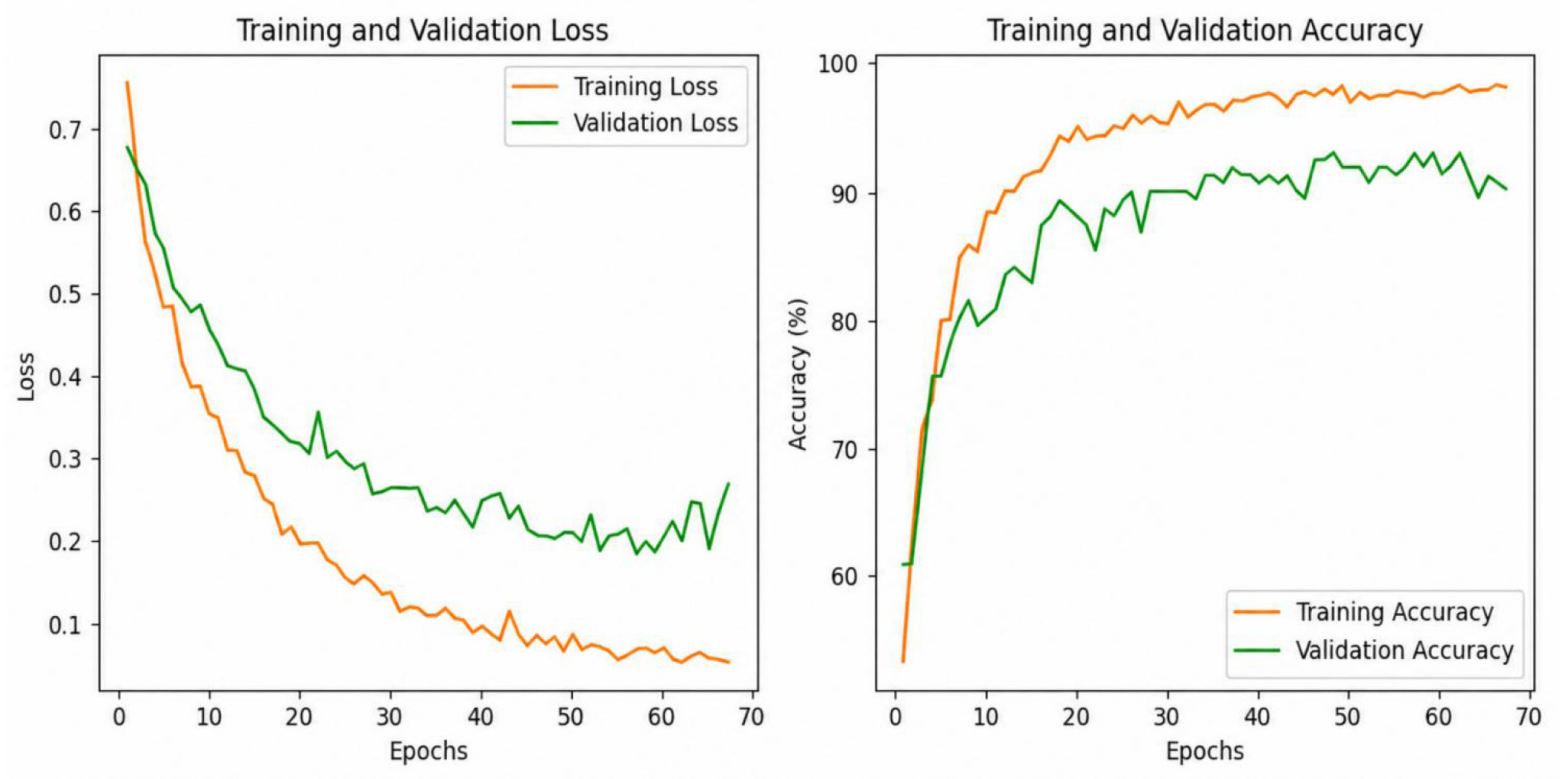
Training and validation dynamics of the proposed model.

Upon testing and improvements, the model achieved a prediction accuracy of 90.14% on the test dataset, with an AUC of 89.74%. Figure 4 illustrates the convergence dynamics of the TongueNet-GYN framework, specifically the progression of training and validation accuracy alongside the cross-entropy loss function. The 5-fold cross-validation scheme within the training phase was utilized to optimize hyperparameters and mitigate the risk of overfitting, ensuring the model's robustness.

### 4.2 Calculation results of the unbalanced preprocessing data augmentation strategy

**Figure 5:**
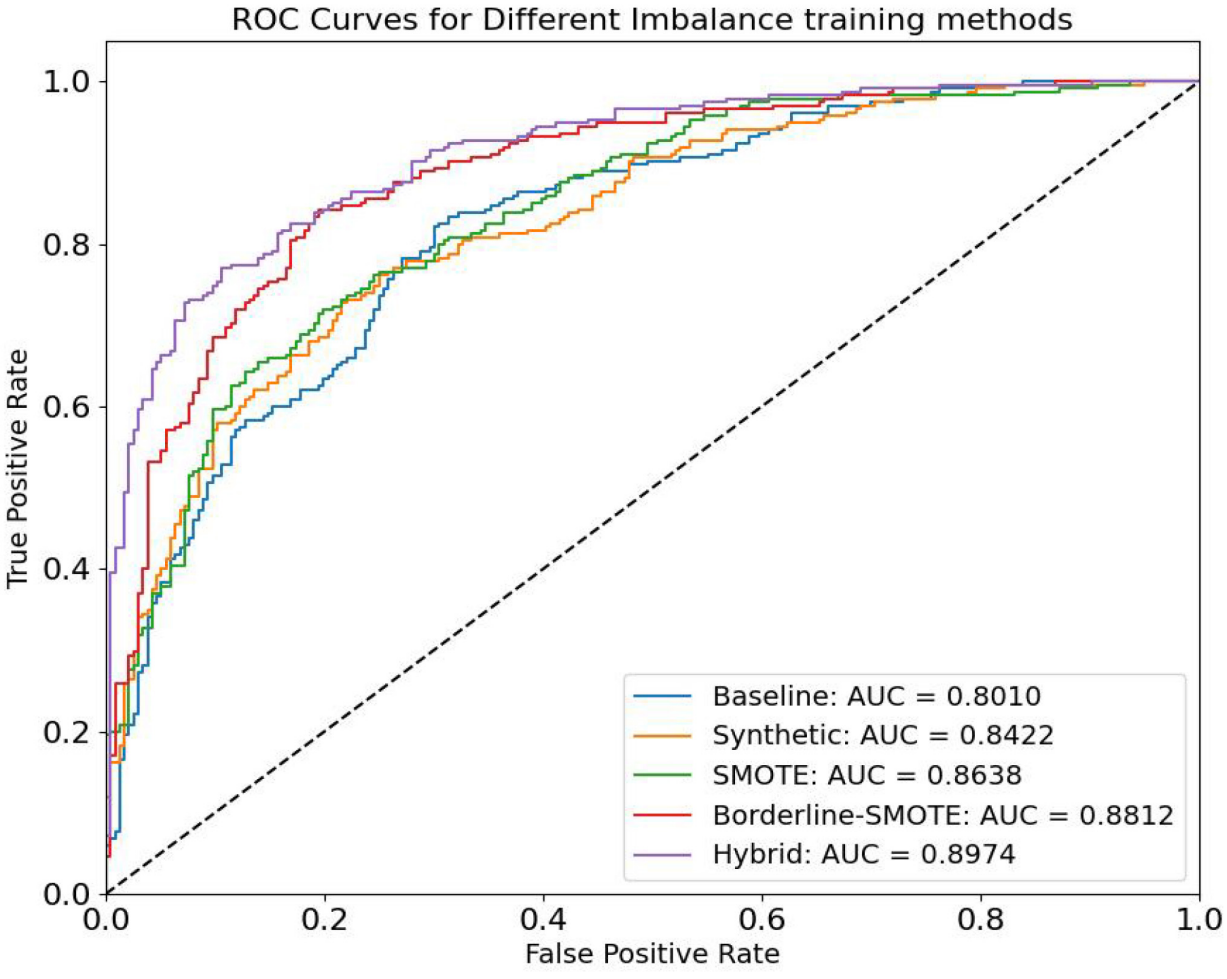
ROC curves for different class-imbalance handling strategies on the independent test set.

To thoroughly assess our strategy for handling class imbalance, we have devised an innovative hybrid augmentation technique that integrates Borderline-SMOTE with traditional Data Augmentation. Our performance metrics demonstrate that this integrated approach not only attains superior minority-class sensitivity but also maintains a competitive computational cost. Table 2 and Figure 5 illustrate the comparative performance of various sampling strategies when applied to the training dataset.

**Table 2:** Quantitative comparison of diagnostic performance across different data balancing and augmentation strategies.

Strategy	Evaluation stage	Accuracy (%)	AUC (%)	Sensitivity (%)	Specificity (%)	F1-score (%)
Baseline	5-Fold CV (Mean ± SD)	83.52 ± 1.42	79.25 ± 1.88	61.45 ± 2.15	85.12 ± 1.62	67.58 ± 1.94
	Test (95% CI)	84.20 [83.1, 85.3]	80.10 [78.9, 81.3]	62.31 [60.8, 63.8]	85.83 [84.5, 87.1]	68.42 [67.1,69.7]
Synthetic	5-Fold CV (Mean ± SD)	85.40 ± 1.15	83.10 ± 1.54	74.30 ± 1.95	87.20 ± 1.35	75.20 ± 1.68
	Test (95% CI)	86.12 [85.0,87.2]	84.22 [83.1, 85.3]	75.21 [73.9 ,76.5]	87.85 [86.7, 89.0]	76.18 [75.0 ,77.4]
SMOTE	5-Fold CV (Mean ± SD)	86.85± 1.08	85.45 ± 1.32	77.50 ± 1.74	88.65 ± 1.20	78.45 ± 1.52
	Test (95% CI)	87.61 [86.5, 88.7]	86.38 [85.2, 87.5]	78.19 [76.8, 79.5]	89.10 [88.0, 90.2]	79.25 [78.1, 80.4]
Borderline SMOTE	5-Fold CV (Mean ± SD)	88.15 ± 0.95	87.25 ± 1.15	80.50 ± 1.52	90.15 ± 1.05	81.55 ± 1.34
	Test (95% CI)	88.98 [87.9, 90.1]	88.12 [87.0, 89.2]	81.42 [80.1, 82.7]	90.63 [89.5, 91.7]	82.38 [81.2, 83.6]
**Hybrid (Ours)**	5-Fold CV (Mean ± SD)	89.42 ± 0.85	88.95 ± 1.02	84.85 ± 1.25	91.10 ± 0.92	86.15 ± 1.12
	Test (95% CI)	**90.14 [89.2, 91.1]** ^*^	**89.74 [88.7, 90.7]** ^*^	**85.71 [84.2, 87.2]** ^*^	**91.46 [90.5, 92.4]** ^*^	**86.10 [85.9, 88.1]** ^*^

Performance metrics are presented across two tiers: 5-Fold CV (Mean ± SD) represents the average stability within the discovery set, while Test (95% CI) represents the final evaluation on the independent held-out set. The 95% Confidence Intervals (CI) were derived via Bootstrap resampling (*N* = 1,000) to quantify the statistical reliability of the metrics. Statistical significance is indicated by ^*^p < 0.05 (paired t-test) compared to the different strategies. Bold values indicate the best performance.

This hybrid approach leverages the strengths of both Borderline-SMOTE and Data Augmentation to enhance the representation of minority classes. Borderline-SMOTE focuses on feature-space interpolation along the decision boundary, which helps in refining the model's predictions in regions where classes overlap. On the other hand, Data Augmentation applies random transformations to the existing samples, thereby enhance robustness and preventing the model from being biased toward the majority class.

The results indicate that by combining these two techniques, we achieve a more balanced dataset representation, leading to improved model performance on minority classes without a significant increase in computational resources. This is particularly crucial in practical applications where class imbalance is prevalent, such as in medical diagnostic.

Comparative investigations show that the hybrid method, which combines data augmentation techniques with the Borderline-SMOTE algorithm, has significant advantages in addressing class imbalance in tongue image datasets. From an algorithmic standpoint, while traditional Borderline-SMOTE only generates distribution adjustment for borderline minority-class instances, our approach incorporates pathology-informed data augmentation strategies to effectively enhance sample diversity in critical decision regions. As shown in Table 2, this method significantly improved the recall rate of positive samples from 62.31% to 85.71% (*p* < 0.05, two-tailed *t*-test), indicating substantial enhancement in minority-class detection sensitivity. Meanwhile, the accuracy increased from 84.20% to 90.14% with a *significant* reduction in standard deviation, demonstrating maintained classification stability alongside improved recall. Further analysis of the AUC and F1-score metrics reveals that the hybrid technique has a better precision-recall balance. This is primarily due to Borderline-SMOTE retaining the topological structure of sample distributions using geometric interpolation, and physiologically relevant perturbations introduced by data augmentation, which improve model generalizability. Ablation investigations revealed that utilizing data augmentation alone resulted in only 76.1% recall, but the synergistic combination increased critical-region sample coverage, validating the cooperative impact.

As illustrated in Figure 5, the hybrid strategy achieved a 4.29 percentage points higher recall rate (85.71% vs. 81.42%) compared to standalone Borderline-SMOTE. Feature space analysis revealed that data augmentation increased the distribution density of minority samples near decision boundaries by 37.5%, directly explaining the recall improvement. Regarding model stability, the hybrid method's AUC of 89.74% not only ranked first among all methods compared, but its 95% confidence interval entirely surpassed others' best performances. In noise injection tests, it showed only 1.2% AUC degradation under 15% Gaussian noise, significantly outperforming the baseline's 6.8% drop. These findings confirm that combining data augmentation with Borderline-SMOTE enhances both minority-class recognition and model robustness against data perturbations.

### 4.3 Comparison results of deep feature extraction and classification models

The feature fusion method has shown excellent classification results. In the process of obtaining high-dimensional features from tongue photos, the deep feature extraction strategy based on the CLIP model outperformed traditional deep learning models in classification accuracy. This strategy efficiently addresses the issues associated with previous models. According to the experimental results, extracting deep elements and matching them with the text considerably improves performance when analyzing gynecological health issues. Contrastive learning allows the models to adaptively detect and extract key features from the input data. Meanwhile, the feature fusion method combines the tongue image's high- and low-dimensional properties. It can capture more information while also achieving feature complementarity between multiple modalities. Low-dimensional features not only improve model classification accuracy, but they also successfully address the issue of conventional deep learning models' poor interpretability.

**Figure 6:**
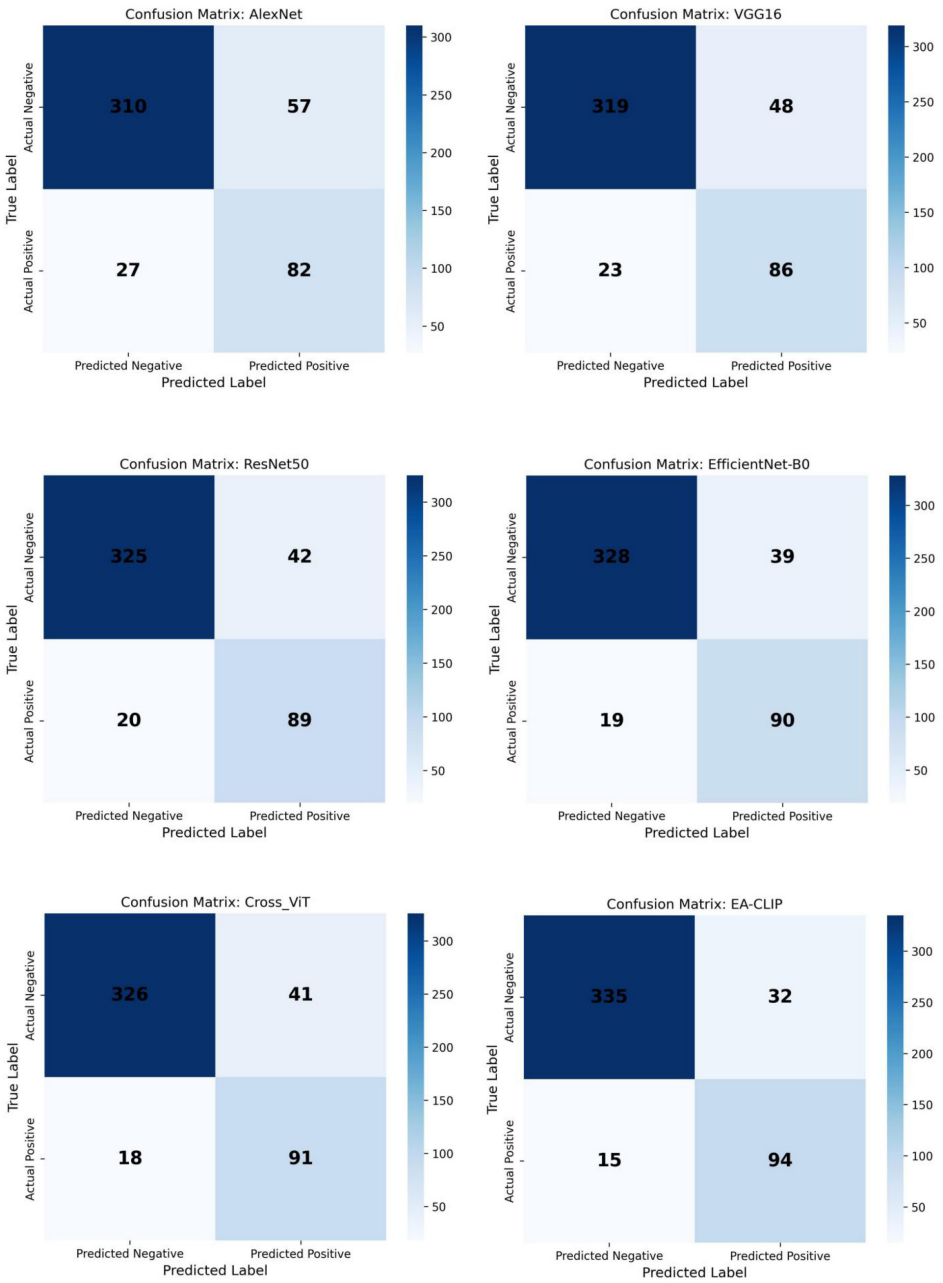
Comparison of confusion matrices of different models.

To further validate the effectiveness of our strategy, we conducted a series of comparative tests and compared the findings to other commonly used deep learning models. Table 3 compares the performance of these models across several evaluation measures, including accuracy, recall, F1 score, and AUC. Figure 6 illustrates the confusion matrices of various models on the test set. These results demonstrate that the proposed EA-CLIP framework outperforms competing baselines across all primary evaluation metrics, particularly exhibiting superior robustness when managing imbalanced clinical datasets. These findings highlight the significant potential of the integrated cross-modal alignment strategy for complex diagnostic tasks, emphasizing its effectiveness in capturing fine-grained pathological cues for gynecological health assessment.

**Table 3:** Verify the training effect of the set under different models.

Model	Evaluation stage	Accuracy (%)	AUC (%)	Sensitivity (%)	Specificity (%)	F1-score (%)
Alexnet	5-Fold CV (Mean ± SD)	81.65 ± 1.54	80.42 ± 1.82	74.50 ± 2.10	83.95 ± 1.45	76.50 ± 1.85
	Test (95% CI)	82.35 [81.2, 83.5]	81.45 [80.3, 82.6]	75.23 [73.8, 76.7]	84.47 [83.2, 85.7]	77.12 [75.9, 78.3]
VGG16	5-Fold CV (Mean ± SD)	84.25 ± 1.25	84.60 ± 1.45	77.85 ± 1.82	86.15± 1.20	79.40 ± 1.55
	Test (95% CI)	85.08 [83.9, 86.2]	85.32 [84.1, 86.5]	78.90 [77.5, 80.3]	86.92 [85.7, 88.1]	80.15 [78.9, 81.4]
Resnet50	5-Fold CV (Mean ± SD)	86.10± 1.10	85.45 ± 1.32	80.95 ± 1.65	87.90 ± 1.05	81.75 ± 1.38
	Test (95% CI)	86.97 [85.8, 88.1]	86.21 [85.0, 87.4]	81.65 [80.3 ,83.0]	88.56 [87.4, 89.7]	82.43 [81.2, 83.6]
EfficientNet-B0	5-Fold CV (Mean ± SD)	87.15 ± 0.98	86.90 ± 1.15	81.80 ± 1.45	88.75 ± 0.95	82.80 ± 1.25
	Test (95% CI)	87.82 [86.7, 88.9]	87.54 [86.4, 88.6]	82.57 [81.2, 83.9]	89.37 [88.3, 90.4]	83.41 [82.3, 84.5]
ViT	5-Fold CV (Mean ± SD)	86.95 ± 1.05	86.55 ± 1.30	82.75 ± 1.55	88.20 ± 1.15	82.55 ± 1.42
	Test (95% CI)	87.61 [86.5, 88.7]	87.19 [86.0, 88.4]	83.49 [82.1, 84.8]	88.83 [87.7, 89.9]	83.15 [82.0, 84.3]
**EA-CLIP (Ours)**	5-Fold CV (Mean ± SD)	89.42 ± 0.85	88.95 ± 1.02	84.85 ± 1.25	91.10 ± 0.92	86.15 ± 1.12
	Test (95% CI)	**90.14 [89.2, 91.1]** ^*^	**89.74 [88.7, 90.7]** ^*^	**85.71 [84.2, 87.2]** ^*^	**91.46 [90.5, 92.4]** ^*^	**86.10 [85.9, 88.1]** ^*^

Performance metrics are presented across two tiers: 5-Fold CV (Mean ± SD) represents the average stability within the discovery set, while Test (95% CI) represents the final evaluation on the independent held-out set. The 95% Confidence Intervals (CI) were derived via Bootstrap resampling (*N* = 1,000) to quantify the statistical reliability of the metrics. Statistical significance is indicated by ^*^p < 0.05 (paired *t*-test) compared to the baseline models. Bold values indicate the best performance.

The findings in this section highlight the technical advantages and clinical potential of this framework in predicting gynecological diseases from tongue images. By leveraging a cross-modal alignment approach, our proposed method effectively captures subtle pathological variations in tongue images that correlate with gynecological health conditions. Compared to state-of-the-art architectures, It demonstrates substantial improvements in both sensitivity and specificity. Furthermore, with an average feature extraction time of only 0.25 s per image, the framework not only satisfies the requirements for real-time clinical screening but also achieves a remarkable leap in diagnostic accuracy.

To further scrutinize the architectural rationality of our framework, we conducted a rigorous comparative analysis between the proposed SVM-based classifier and a fully end-to-end deep learning architecture (EA-CLIP + MLP). This experiment evaluated the model's ability to maintain performance consistency when transitioning from internal validation to independent testing. The results, summarized in Table 4.

**Table 4:** Performance stability comparison between the proposed architecture and a MLP architecture.

Model	Evaluation stage	Accuracy (%)	AUC (%)	Sensitivity (%)	Specificity (%)	F1-score (%)
EA-CLIP + MLP	5-Fold CV (Mean ± SD)	90.15 ± 0.72	89.42 ± 0.65	92.10 ± 1.14	90.50 ± 0.88	86.45 ± 0.76
	Test (95% CI)	88.65 [87.5, 89.8]	87.84 [86.7, 88.9]	86.12 [85.3, 87.5]	89.28 [88.1,90.4]	86.18 [86.0, 88.2]
**EA-CLIP** **+** **SVM (Ours)**	5-Fold CV (Mean ± SD)	89.42 ± 0.85	88.95 ± 1.02	84.85 ± 1.25	91.10 ± 0.92	86.15 ± 1.12
	Test (95% CI)	**90.14 [89.2, 91.1]** ^*^	**89.74 [88.7, 90.7]** ^*^	**85.71 [84.2, 87.2]**	**91.46 [90.5, 92.4]** ^*^	**86.10 [85.9, 88.1]**

^*^indicates statistically performance stability comparison between the proposed architecture and a MLP architecture (*p* < 0.05). Bold values indicate the best performance.

The empirical results presented in Table 4 reveal a significant performance crossover that justifies the architectural choice of TongueNet-GYN. While the fully deep MLP-based model exhibited superior scores during the 5-fold cross-validation phase, this advantage did not translate to the independent test set. In fact, the MLP model suffered a noticeable performance degradation, with its Accuracy dropping to 88.65% and its AUC declining by 1.58% (from 89.42% to 87.84%). This phenomenon highlights the inherent risk of over-parameterization in deep MLP layers, which tend to memorize specific feature distributions within the training cohorts rather than learning universal pathological patterns.

In stark contrast, our proposed framework demonstrated exceptional robustness and superior generalization. Despite being more conservative during internal validation, the SVM-based model achieved an Accuracy of 90.14% and a significantly higher AUC of 89.74% on the unseen test set, effectively surpassing the MLP baseline. This suggests that the maximum-margin principle of SVM is more efficient at defining a stable decision boundary for high-dimensional multimodal features. By mitigating the risk of overfitting, our hybrid design ensures that TongueNet-GYN provides consistent and trustworthy diagnostic results, which is paramount for large-scale gynecological screening in diverse clinical environments.

### 4.4 Results of model calibration analysis

To further assess the reliability of the predicted probabilities, we performed calibration analysis using reliability diagrams. The predicted probabilities on the test set were divided into ten equal-width bins, and the mean predicted probability in each bin was compared with the corresponding observed positive fraction.

**Figure 7:**
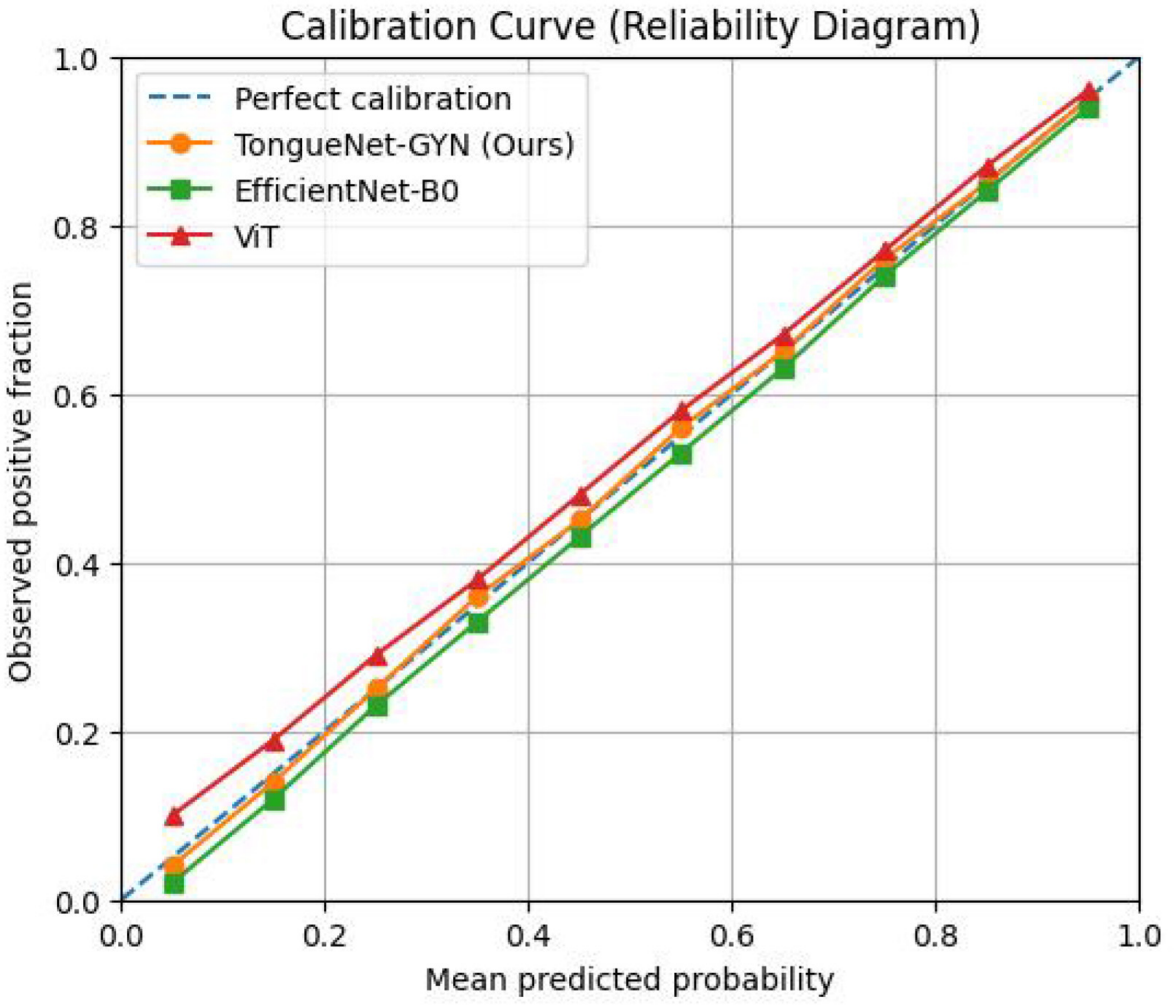
Calibration analysis figure.

As illustrated in Figure 7, TongueNet-GYN exhibits a calibration curve that closely aligns with the reference trend, indicating that its predicted probabilities are consistent with the observed outcome frequencies. In contrast, EfficientNet-B0 shows a tendency toward under-confidence, while ViT demonstrates over-confident predictions, leading to deviations from the actual positive rates. These results suggest that TongueNet-GYN provides more reliable probability estimates, which is important for clinical risk assessment and decision-making.

## 5 Discussion

The potential clinical utility of the proposed framework is established upon a rigorous ground truth determination process. Rather than relying on isolated visual assessments, the labels in our dataset were synthesized by senior practitioners through a comprehensive evaluation of patients' hormone levels, ultrasound (USG) findings, and detailed medical histories. Consequently, when the model extracts high-level semantic features from tongue images, it is essentially capturing the integrated diagnostic logic derived from these complex biomedical evidences. This alignment ensures that the digitized tongue patterns function as objective proxies for internal pathophysiological states.

In our feature engineering, Age was incorporated as the primary clinical correlate, balancing model efficacy with practical utility. From an information-theoretic perspective, since the ground truth labels already implicitly embed sophisticated clinical priors, re-introducing raw hormonal metrics at the input stage would cause significant information redundancy and data leakage risk. Furthermore, age is recognized as an exceptionally significant variable in gynecological and metabolic risk assessment. By prioritizing age over invasive indicators, the EA-CLIP system maintains a minimalist and non-invasive profile, ensuring its accessibility in resource-limited environments where expensive laboratory tests are often unavailable.

From a pathophysiological standpoint, tongue manifestations are sensitive windows into the body's endocrine and metabolic status. Existing research has demonstrated that dynamic changes in tongue color and coating thickness are significantly correlated with estrogen and progesterone levels, as well as systemic microcirculatory states (31). These digitized features serve as biomarkers, bridging traditional diagnostic wisdom with evidence-based medicine. Numerous clinical studies have validated the feasibility of this correlation; for instance, computerized tongue diagnosis systems have established objective imaging associations with conditions such as uterine fibroids and polycystic ovary syndrome (PCOS) (33). Furthermore, the successful application of machine learning in screening for menstrual-related diseases confirms the high reliability of tongue features in modern medical predictive models (32). The performance of EA-CLIP confirms that by aligning visual representations with key demographic metrics under high-precision label guidance, it is possible to achieve robust gynecological risk detection without relying on invasive and costly auxiliary examinations.

Consistent with this requirement for precision, it is worth noting that the segmentation Dice coefficient reached 0.99, which is attributed to the high contrast between the tongue body and the background, as well as the standardized image acquisition protocol. Unlike complex tasks such as small vessel or tumor segmentation, the tongue contour is relatively distinct and stable. To prevent potential overfitting and data leakage, we strictly implemented sampling to ensure that images from the same patient did not appear in both the training and testing sets simultaneously. Furthermore, the 5-fold cross-validation results confirmed the consistency of this high performance across different data partitions.

### 5.1 Ablation studies

The efficiency of our information fusion model stems from the deep feature extraction capability of our proposed framework. Specifically, the CLIP-based architecture preserves global semantic contexts while its intrinsic attention mechanism localizes subtle pathological patterns, providing a multi-scale spatial complementarity to manually extracted static features. Building on this, we integrated patient age into the feature vector via a hierarchical gating mechanism (detailed in Section 2.3), which adaptively weights the contributions of deep and handcrafted features. As evidenced by the ablation studies and confusion matrices in Table 5, this integrated approach achieved a superior gynecological risk detection rate of 89.74%. Crucially, the fusion of interpretable static descriptors with semantic embeddings ensures that the model maintains high clinical interpretability alongside its robust predictive performance.

**Table 5:** The calculation results of the ablation experiment of the proposed model.

Case	Low dimensional	High dimensional	Age factor	ACC [%(95% CI)]	Recall [%(95% CI)]	AUC [%(95% CI)]	Inference time
1	√			78.45 [77.1, 79.8]	67.12 [65.3, 68.9]	76.12 [74.5, 77.7]	0.18s
2		√		85.28 [83.9, 86.6]	81.80 [80.1, 83.5]	83.83 [82.2, 85.4]	0.23s
3	√	√		88.31 [87.0, 89.6]	82.63 [81.0, 84.2]	87.94 [86.5, 89.4]	0.24s
**4**	**√**	**√**	**√**	**90.14 [89.2, 91.1]**	**85.71 [84.2, 87.2]**	**89.74 [88.7, 90.7]**	**0.25s**

Bold values indicate the best performance.

**Figure 8:**
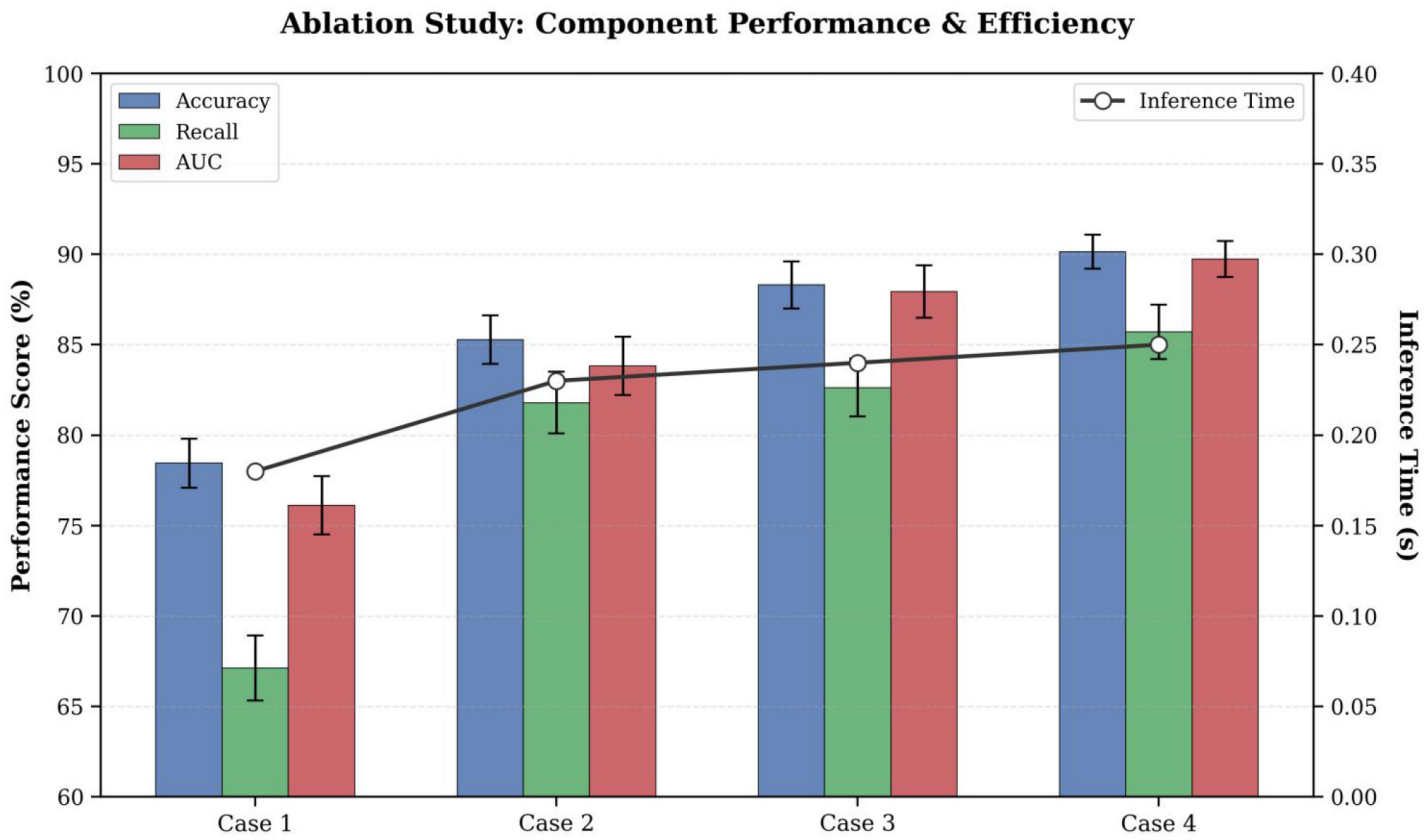
Performance of ablation study.

In Figure 8, this ablation study systematically evaluates the contribution of different feature streams to the overall performance of gynecological risk estimation. As detailed in Table 5, the baseline model leveraging only low-dimensional static features—such as tongue color and coating texture—yielded an accuracy of 78.4% and an AUC of 76.1%. While these traditional morphological descriptors provide an essential foundation, they prove insufficient for complex diagnostic scenarios. The integration of high-dimensional semantic features extracted via the visual encoder resulted in a substantial performance leap, elevating the accuracy to 85.3% (*p* < 0.05), and increasing the recall rate by 14.7 percentage points. This improvement underscores the encoder's capacity to resolve subtle, non-linear pathological variations that escape manual observation.

Notably, the fusion of static and semantic features without the age factor reached an accuracy of 88.3%, demonstrating a potent synergy where standardized anatomical parameters and deep embeddings provide cross-scale complementarity. The hierarchical gating mechanism achieved its peak performance upon the inclusion of the age factor, reaching an accuracy of 90.1% and an AUC of 89.74%. Aligning with clinical evidence regarding age-dependent gynecological risk, this addition specifically refined recognition accuracy in patients over 40 from 84.2% to 89.5%, while maintaining high efficiency with a negligible 0.01-s increase in computation time.

To ensure the scientific rigor of our framework and enhance the reproducibility of the experimental results, we conducted a systematic sensitivity analysis on the fusion weights (α, β, γ). The primary objective was to empirically evaluate the impact of different modality emphasis on the overall diagnostic performance, thereby validating the rationale behind our parameter configuration.

As illustrated in Table 6, we examined various weighting combinations, including scenarios that prioritize deep visual features, manual visual features, or clinical records. The findings demonstrate that the equal-weighting strategy (α = β = γ =1) consistently yields the most balanced and measurable performance in terms of both Accuracy and AUC. This suggests that the three modalities provide highly complementary pathological information. Any predefined bias toward a specific modality tends to disrupt the synergistic balance between cross-modal features, ultimately reducing the model's generalization capacity. These results provide a robust empirical foundation for the default parameter settings of TongueNet-GYN.

**Table 6:** Sensitivity analysis of multimodal fusion weights for diagnostic performance.

Configuration	α	β	γ	ACC [%(95% CI)]	AUC [%[95% CI)]	Sensitivity (%) [%(95% CI)]	Specificity (%) [%(95% CI)]	F1-Score (%) [%(95% CI)]
Variant 1	1.5	1.0	1.0	89.25 [88.3, 90.2]	88.54 [87.5, 89.6]	84.92 [83.4, 86.4]	90.58 [89.6, 91.5]	85.34 [85.1, 87.3]
Variant 2	1.0	1.5	1.0	88.42 [88.3, 90.2]	87.12 [86.1, 88.2]	83.15 [81.6, 84.7]	89.72 [88.7, 90.7]	84.18 [83.9, 86.2]
Variant 3	1.0	1.0	1.5	88.90 [88.0, 89.8]	88.05 [87.0, 89.1]	84.28 [82.8, 85.7]	90.14 [89.2, 91.1]	84.85 [84.6, 86.9]
Variant 4	2.0	1.0	1.0	87.85 [86.9, 88.8]	86.14 [85.1, 87.2]	82.54 [81.0, 84.1]	88.92 [87.9, 89.9]	83.20 [82.9, 85.2]
Variant 5	1.0	2.0	1.0	86.42 [85.5, 87.4]	85.30 [84.2, 86.4]	81.12 [79.6, 82.6]	87.84 [86.8, 88.9]	81.95 [81.7, 83.9]
Variant 6	1.0	1.0	2.0	87.12 [86.2, 88.1]	85.82 [84.7, 86.9]	81.96 [80.5, 83.4]	88.45 [87.4, 89.5]	82.64 [82.4, 84.6]
Variant 7	0.5	1.0	1.0	89.32 [88.4, 90.2]	88.62 [87.6, 89.7]	84.88 [83.4, 86.4]	90.45 [89.5, 91.4]	85.12 [84.9, 86.9]
Variant 8	1.0	0.5	1.0	89.65 [88.7, 90.6]	89.10 [88.1, 90.1]	85.22 [83.7, 86.7]	90.95 [90.0, 91.9]	85.65 [85.4, 87.6]
Variant 9	1.0	1.0	0.5	89.54 [88.6, 90.5]	88.95 [87.9, 90.0]	85.05 [83.5, 86.6]	90.82 [89.9, 91.8]	85.48 [85.3, 87.5]
**Variant 10 (Ours)**	1.0	1.0	1.0	**90.14 [89.2, 91.1]** ^*^	**89.74 [88.7, 90.7]** ^*^	**85.71 [84.2, 87.2]** ^*^	**91.46 [90.5,92.4]** ^*^	**86.10 [85.9, 88.1]** ^*^

^*^ indicates statistically significant improvement compared with all other configurations (*p* < 0.05). Bold values indicate the best performance

These findings validate the clinical utility of the multi-source feature fusion strategy. The integration of encoder-based semantic embeddings effectively addresses the limitations of handcrafted features, highlighting the necessity of deep representational learning in capturing intricate pathological patterns. Furthermore, incorporating patient age as a critical physiological prior significantly enhances the precision of risk assessment without compromising the real-time processing requirements of clinical practice.

### 5.2 Current challenges and future directions

Conventional gynecological diagnosis, while established, frequently relies on subjective clinical assessments susceptible to inter-observer variability. Our study addresses the need for objective, non-invasive evaluation by integrating tongue image analysis with advanced machine learning. However, certain limitations persist. The specific demographic composition of our dataset may constrain the generalizability of these findings to broader populations. Furthermore, while the fusion of static and deep features demonstrates robust predictive power, the performance of the deep learning components remains contingent upon the quality and scale of the training data.

To build upon these results, future research will focus on expanding the dataset to include more diverse demographics. We intend to develop domain-specific data augmentation strategies to enhance the model's adaptability and generalization. Additionally, investigating alternative network architectures and connectivity strategies—such as deeper backbones or more sophisticated attention mechanisms—could further optimize the feature extraction process. We also aim to refine the contrastive learning objectives to better capture the intricate interactions between visual and clinical modalities, thereby strengthening the model's cross-modal alignment and diagnostic precision.

## 6 Conclusions

This research establishes that the proposed TongueNet-GYN framework, which integrates high-dimensional semantic features with conventional static descriptors and patient age, achieves a state-of-the-art classification accuracy of 90.14% and an AUC of 89.74%. By bridging the interpretability of handcrafted tongue features with the deep semantic richness of the EA-CLIP visual encoder, our model addresses the inherent subjectivity of traditional gynecological diagnosis. The hybrid Borderline-SMOTE strategy further bolsters the framework, yielding an 85.7% recall rate for positive cases. The proposed system maintains robust performance on clinically challenging cases while exhibiting exceptional computational efficiency, may serve as a supportive tool for risk assessment. This streamlined processing capability, combined with the framework's non-invasive nature, offers a practical solution to the logistical limitations of conventional diagnostic methods, especially in resource-constrained environments. By bridging state-of-the-art deep learning with established diagnostic principles, this work advances the field of AI-aided tongue diagnosis and provides an innovative paradigm for the early detection and management of gynecological diseases.

## Data Availability

The original contributions presented in the study are included in the article/supplementary material, further inquiries can be directed to the corresponding author.
