# Supplementary material for: TongueNet-GYN: a multimodal deep learning framework for non-invasive gynecological disease screening in digital public health
Source: Front Public Health. 2026 Jun 23;14:1854215. doi: 10.3389/fpubh.2026.1854215 (PMC13337768; doi:10.3389/fpubh.2026.1854215)
Supplement: Supplementary file 1 [file Data_Sheet_1.pdf]

Supplementary Material

1 Supplementary Table S1

Category	Variables	Risk Group	Control Group	P - Value
Demographics	Age (years)	34.21 ± 8.45	28.63 ± 7.18	**< 0.001**
	BMI (kg/m^2)	23.85 ± 3.42	22.92 ± 2.56	0.064
	Family History of GYN Diseases(n%)	312 (24.7%)	246 (12.9%)	0.015
Clinical Priors & Labels	Hormonal Imbalance (E2/P/T) (n%)	856 (67.8%)	52 (2.7%)	**< 0.001**
	Abnormal Pelvic USG Findings (n%)	942 (74.6%)	28 (1.5%)	**< 0.001**
	Menstrual Cycle Irregularity (n%)	785 (62.2%)	148 (7.8%)	**< 0.001**
	Hemoglobin (g/L)	118.5 ± 12.4	126.2 ± 10.8	0.058

Clinical Subtypes (Risk Group Only)	Uterine Fibroids (n%)	422 (33.4%)	-	-
	Polycystic Ovary Syndrome (PCOS)(n%)	548 (43.4%)	-	-
	Other Gynecological Inflammations (n%)	292 (23.1%)	-	-
Tongue Manifestations	Dark Red or Purple Tongue Body (n%)	642 (50.9%)	112 (5.9%)	**< 0.001**
	Thick and Greasy Tongue Coating (n%)	588 (46.6%)	204 (10.7%)	0.004
	Sublingual Varicosities (n%)	425 (33.7%)	86 (4.5%)	0.012
	Marginal Teeth Marks (n%)	314 (24.9%)	135 (7.1%)	0.021

Table Note 1: The study cohort was systematically recruited from Jiangsu Kunshan Hospital under a rigorous quality control framework. Eligible participants included females aged 18 – 65 with laboratory-confirmed gynecological conditions (e.g., PCOS, uterine fibroids), while strict exclusion criteria were enforced to eliminate confounding factors such as pregnancy, acute infections, oral

lesions, and dietary-induced tongue discolouration. This meticulous selection process ensures the integrity of the multimodal screening model by minimizing interference with tongue image analysis.

Table Note 2: The diagnostic Ground Truth was established through a rigorous synthesis of objective clinical evidence, moving beyond simple visual assessment. A panel of three senior experts, each with over ten years of integrated experience in Gynecology and Traditional Chinese Medicine, performed the labeling by cross-referencing participants' biochemical profiles (serum estradiol, progesterone, and testosterone levels) with pelvic ultrasound findings. This multi-modal evidence served as a high-precision clinical prior to ensure that each categorical label reflected the patient's definitive pathophysiological status. To further guarantee data reliability, a formal adjudication mechanism was employed, where any diagnostic divergences were resolved by a third senior specialist, ensuring maximum accuracy and consistency for the model's learning objectives.

2 Eligibility Criteria for Study Participants

Inclusion Criteria:

Target Population: Females aged 18 to 65 years who underwent gynecological examinations at Jiangsu Kunshan Hospital of Integrated Traditional Chinese and Western Medicine.

Diagnostic Confirmation (Risk Group): Participants must have a laboratory-confirmed diagnosis of at least one target gynecological condition supported by objective clinical evidence:

(1) **Biochemical Evidence**: Abnormal serum endocrine profiles, including but not limited to elevated Serum Testosterone (> 2.6 nmol/L) or an **LH/FSH** ratio ≥ 2.

(2) **Imaging Evidence**: Positive findings in pelvic or transvaginal ultrasound (USG), such as the presence of uterine myomas, ovarian cysts, or polycystic ovarian morphology.

Informed Consent: Participants who voluntarily signed the written informed consent and agreed to the standardized acquisition of digital tongue images and the use of their de-identified clinical data for research purposes.

Exclusion Criteria:

(1) **Physiological & Temporary Factors**: Currently pregnant or lactating, or in the active menstrual period at the time of imaging, to minimize the interference of acute physiological hormonal fluctuations on tongue manifestations.

(2) **Systemic Conditions**: History of malignant tumors, severe cardiovascular, hepatic, or renal insufficiency, or acute systemic infectious diseases that may systemicly alter the tongue appearance.

(3) **Oral & Dietary Interference**: Presence of primary oral diseases (glossitis, severe dental plaque, or oral candidiasis) that significantly obscure or alter the tongue surface characteristics. Consumption of food, beverages, or medications known to cause tongue coating staining (coffee, strong tea, or candy with artificial dyes) within 2 hours prior to image acquisition.

(4) **Data Quality Control**: Tongue images characterized by severe motion blur, inappropriate exposure (over-exposed or under-exposed), or incomplete visibility of the tongue body.

Images were subjected to a rigorous multi-stage quality screening process and were excluded if they met any of the following technical criteria:

(1) Severe Motion Blur and Defocus, defined as images where the structural edges of lingual papillae or fissures are indistinct, specifically those with a Laplacian variance focus score below a calibrated threshold of 100 to ensure high-frequency texture integrity.

(2) Inappropriate Exposure, including over-exposed images where more than 5% of the tongue body surface pixels reach a saturation value of 255, or under-exposed images with a mean pixel intensity below 40, rendering color differentiation unreliable.

(3) Incomplete Visibility or Occlusion, where more than 10% of the tongue body is obscured by lips, teeth, or medical instruments, or where the tongue root is not fully extended.

(4) Specular Artifacts, characterized by significant glare or light reflection spots covering more than 15% of the central diagnostic region, which could potentially distort the model's attention mechanisms and lead to false feature extraction.
